# Recent advances in drug delivery of celastrol for enhancing efficiency and reducing the toxicity

**DOI:** 10.3389/fphar.2024.1137289

**Published:** 2024-02-16

**Authors:** Yuan Sun, Chengen Wang, Xiaoguang Li, Jun Lu, Maolin Wang

**Affiliations:** ^1^ Clinical Research Center, The First Affiliated Hospital of Shantou University Medical College, Shantou, China; ^2^ Department of Minimally Invasive Tumor Therapies Center, Beijing Hospital, National Center of Gerontology, Institute of Geriatric Medicine, Beijing, China; ^3^ State Key Laboratory of Southwestern Chinese Medicine Resources, School of Pharmacy, Chengdu University of Traditional Chinese Medicine, Chengdu, China; ^4^ Department of Physiology, School of Basic Medical Sciences, Shenzhen University Health Sciences Center, Shenzhen, China

**Keywords:** celastrol, enhancing efficiency, reducing toxicity, monomeric ingredient, traditional Chinese medicines

## Abstract

Celastrol is a quinone methyl triterpenoid monomeric ingredient extracted from the root of *Tripterygium wilfordii*. Celastrol shows potential pharmacological activities in various diseases, which include inflammatory, obesity, cancer, and bacterial diseases. However, the application prospect of celastrol is largely limited by its low bioavailability, poor water solubility, and undesired off-target cytotoxicity. To address these problems, a number of drug delivery methods and technologies have been reported to enhance the efficiency and reduce the toxicity of celastrol. We classified the current drug delivery technologies into two parts. The direct chemical modification includes nucleic acid aptamer–celastrol conjugate, nucleic acid aptamer–dendrimer–celastrol conjugate, and glucolipid–celastrol conjugate. The indirect modification includes dendrimers, polymers, albumins, and vesicular carriers. The current technologies can covalently bond or encapsulate celastrol, which improves its selectivity. Here, we present a review that focalizes the recent advances of drug delivery strategies in enhancing the efficiency and reducing the toxicity of celastrol.

## 1 Introduction

Traditional Chinese medicines (TCMs) were the primary therapeutic method used in ancient times by the Chinese to resist diseases, and their bioactive ingredients extracted from herbal plants exhibit various therapeutic properties in a series of diseases, which include inflammatory, obesity, cancer, and bacterial diseases (Dong et al., 2019). Drug designing from natural plants has received more attention recently due to safety, potential effects, and other properties (Hansen et al., 2011). Celastrol, also named tripterine, is one of the most plentiful active molecules extracted from the root of *Tripterygium wilfordii*. In the past years, celastrol has been reported as an ingredient of TCM that can be employed to combat a series of diseases, which include inflammatory, cancer, auto-immune, obesity, and neurodegenerative diseases, by regulating diverse targets (Ng et al., 2019). The possible effective mechanism is that celastrol could exert massive therapeutic effects by influencing the activities of a broad range of targets. Up to now, of all the reports and experiments of celastrol that were employed in the preclinical stage, the outstanding bioactivities and excellent experimental data indisputably support the growing interest in celastrol as one of the top five natural active compounds in the 21st century (Corson and Crews, 2007).

One area of research on celastrol has focused on its potential anti-inflammatory effects. Inflammation is a key factor in many chronic diseases, such as diabetes, cancer, and neurodegenerative disorders. Studies have shown that celastrol has potent anti-inflammatory effects by targeting multiple signaling pathways involved in inflammation, such as the NF-κB, JAK/STAT, and MAPK pathways (Cascão et al., 2017). By inhibiting these pathways, celastrol can reduce inflammation in various tissues and organs, providing a potential therapeutic option for inflammatory diseases (Venkatesha and Moudgil, 2016), which include rheumatoid arthritis (RA) (Yang et al., 2022), osteoarthritis (OA) (Feng et al., 2020), allergic asthma (AA) (Kim et al., 2009), and Crohn’s disease (CD) (Pinna et al., 2004). Celastrol primarily impedes the generation of pro-inflammatory cytokines, nuclear factor-kappa B (NF-κB), and adhesion molecules (Joshi et al., 2016), which all take part in inflammation processes. Celastrol also binds the nuclear receptor Nur77 to inhibit inflammation by autophagy (Hu et al., 2017). Besides, celastrol exerts neuroprotection in Parkinson’s disease (PD) by activating mitophagy to degrade impaired mitochondria and further inhibit dopaminergic neuronal apoptosis (Lin et al., 2019). Celastrol acts as a small molecule inhibitor and has been shown to inhibit Hsp90-Cdc37 complexes in Alzheimer’s disease (AD). It was found that celastrol experiments resulted in modest changes in Tau levels (Blair et al., 2014).

Another area of research on celastrol has focused on its potential to treat obesity. Obesity is a major risk factor for many chronic diseases, such as diabetes and cardiovascular diseases. Studies have shown that celastrol can reduce body weight, improve glucose metabolism, and reduce inflammation in obese mice. Celastrol achieves these effects by targeting the hypothalamus, the part of the brain that regulates appetite and energy metabolism. Celastrol has also been shown to reduce food intake and increase energy expenditure in obese mice, suggesting that it may be a promising treatment for obesity (Liu et al., 2015).

Furthermore, celastrol has been studied for its potential anticancer effects. Studies have shown that celastrol can induce apoptosis (programmed cell death) in cancer cells by targeting multiple signaling pathways. Celastrol can also inhibit cancer cell proliferation, invasion, and angiogenesis (the formation of new blood vessels that feed tumors). In addition, celastrol has been shown to sensitize cancer cells to chemotherapy and radiation therapy, making them more susceptible to these treatments. These findings suggest that celastrol shows potential as a therapeutic agent for cancer treatment (Shi et al., 2020). Up to date, a large number of studies have reported that celastrol exhibits anticancer potential properties in diverse cell lines and animal models, such as in colorectal/colon cancer (Ni et al., 2019), breast cancer (Kim et al., 2011), renal cell carcinoma (Zhang et al., 2021), liver cancer (Du et al., 2020), ovarian cancer (Li et al., 2019), prostate cancer (Ji et al., 2015), lung cancer (Chen et al., 2022), and gastric cancer (Lei et al., 2022). The possible anticancer mechanisms of celastrol are the induction of cell apoptosis, cell cycle arrest, and the suppression of cell propagation (Ng et al., 2019). The detailed mechanism of celastrol in apoptosis includes that celastrol can inhibit the expression of BCL-2 and elevate BAX and cleaved-caspase 3. Furthermore, celastrol may downregulate the miR-21 expression and partially inhibit the PI3K/AKT/GSK-3β pathway (Ni et al., 2019). Also, celastrol has the ability to improve apoptosis of tumor cells by the modulation of reactive oxygen species (ROS) (Chen et al., 2020b) and NF-κB signaling (Ni et al., 2014). Therefore, all the above properties make celastrol a prospective molecule for anticancer clinical application.

Although celastrol exhibits prospective therapeutic properties in a series of diseases, it is extremely restricted by severe side effects in clinical application. However, the potential toxicity of celastrol has also been a subject of concern. Studies have reported that celastrol can exhibit cytotoxicity, hepatotoxicity, and even neurotoxic effects at high concentrations or with prolonged exposure, highlighting the importance of carefully evaluating its therapeutic potential and determining optimal dosages for specific applications (Yang et al., 2006). The main reasons are poor water solubility, low bioavailability, and off-target effects (Shi et al., 2020). To address these drawbacks, the majority of solutions is to develop a delivery system that can carry celastrol to the target sites. Hence, we present a review that focalizes the recent advances of delivery system strategies of celastrol in enhancing the efficiency and reducing the toxicity of celastrol.

## 2 Direct chemical modification delivery system

Direct chemical modification delivery systems are strategies used to improve the pharmacokinetics, stability, and bioavailability of therapeutic molecules, such as nucleic acids or peptides, by covalently attaching specific chemical groups or moieties to these molecules. This modification alters their physicochemical properties, which can help overcome challenges related to drug delivery, such as rapid degradation, immune system recognition, or poor cellular uptake. Examples of direct chemical modification methods include PEGylation (attachment of polyethylene glycol), lipidation (attachment of lipids), and glycosylation (attachment of sugars). These modifications can enhance the therapeutic potential and safety of a drug candidate by optimizing its circulation time, stability, and distribution within the body. We classified the direct chemical modification of celastrol into nucleic acid aptamer–celastrol conjugate (NACC), nucleic acid aptamer–dendrimer–celastrol conjugate, and glucolipid–celastrol conjugate. A detailed description of the different modifications for celastrol is shown in Table 1.

**TABLE 1:** Detailed description of different modifications for celastrol.

Modification	Classification	Disease	Aim	References
Direct	Nucleic acid aptamer	Pancreatic cancer	Enhance the antitumor efficacy and reduce the toxicity	Liu (2017)
	Nucleic acid aptamer–dendrimer	Colorectal cancer	Enhance the antitumor efficacy and reduce the toxicity	Ge et al. (2020)
	Glucolipid	Breast cancer	Enhance the antitumor efficacy	Zhao et al. (2018)
Indirect	Dendrimer	Inflammatory	Enhance the anti-inflammatory efficacy	Boridy et al. (2012)
	Polymer	Prostate cancer	Enhance the antitumor efficacy and reduce the toxicity	Sanna et al. (2015)
	Albumin	Mesangioproliferative glomerulonephritis	Enhance the activity	Guo et al. (2017)
	Albumin	Rheumatoid arthritis	Enhance the safety and reduce the side effects	Gong et al. (2019)
	Vesicular liposome	Hepatocellular carcinoma	Reduce the damage to liver tissue	Chen et al. (2020a)
	Vesicular exosome	Lung cancer	Enhance the antitumor efficacy	Aqil et al. (2016)
	Vesicular nanostructured lipid carrier	Skin disease	Prolong release property and increase the celastrol accumulation	Chen et al. (2012)
	Vesicular bilosome	Arthritis	Enhance anti-inflammatory effect	Yang et al. (2019)
	Vesicular phytosome	—	Enhance oral bioavailability	Freag et al. (2018)

Aptamers are single-stranded oligonucleotides that can specifically identify and integrate to target proteins by distinct secondary or tertiary structures (Keefe et al., 2010). The nucleic acid aptamer modification facilitates the selective accumulation of the conjugated molecule in target rather than normal tissues, subsequently remarkably enhancing efficiency and reducing toxicity (Li et al., 2017).

**FIGURE 1:**
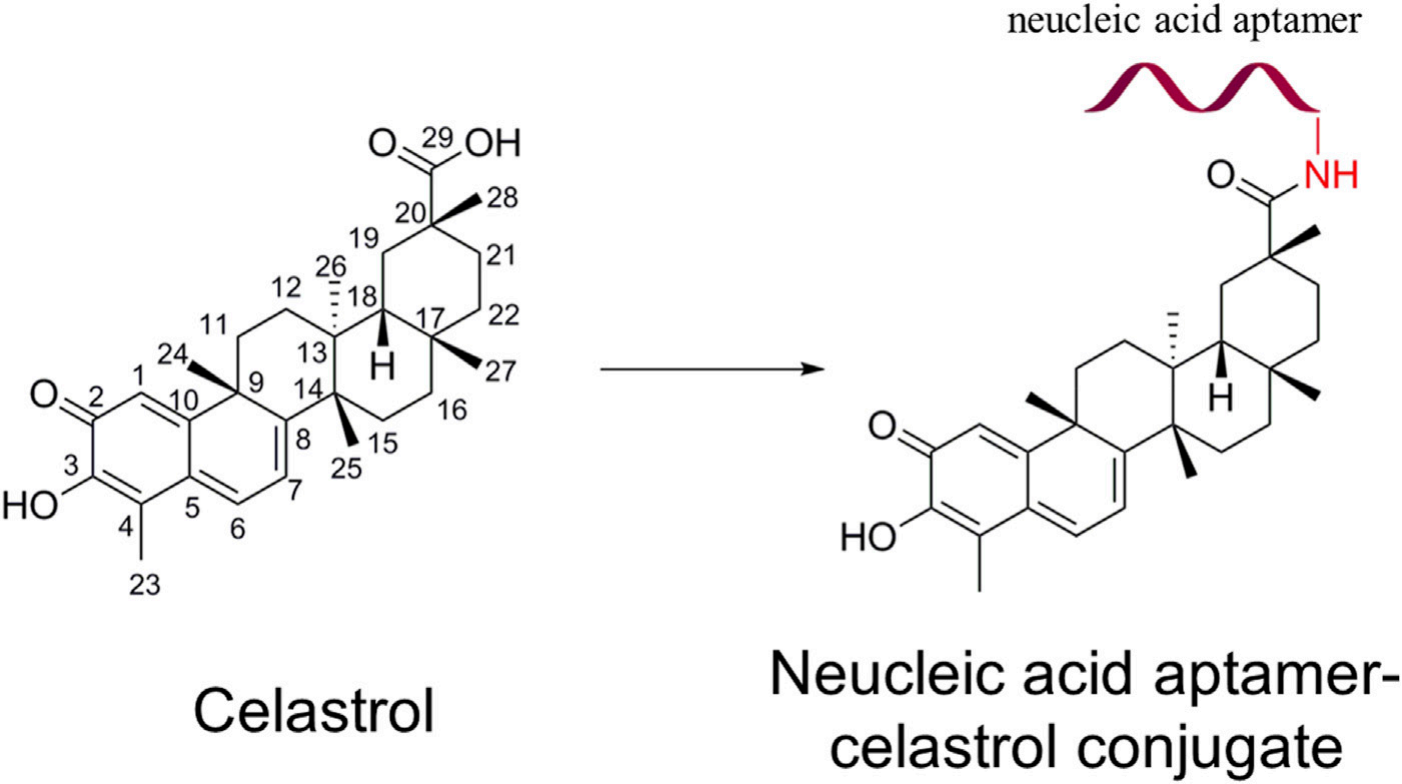
Structure of celastrol and nucleic acid aptamer–celastrol conjugate. The red ribbon represents the nucleic acid aptamer.

Li et al. (2017) developed an NACC to facilitate the conjugated celastrol selectively targeting pancreatic cancer (PC) cells achieve higher antitumor activity and less liver toxicity, as shown in Figure 1. After the direct chemical modification, the NACC exhibited notably higher water solubility than celastrol. The reported solubility of celastrol in water is not more than 0.014 mg/mL (approximately 30 μM) (Bai et al., 2021), while the solubility of the NACC in water is at least greater than 17.8 mg/mL (2 mM). Furthermore, the anticancer efficacy of NACC was significantly higher than that of celastrol *in vivo* and *in vitro*. The IC_50_ value of the NACC on PANC-1 cells was 200 nM (24 h) and 70 nM (48 h), which are significantly lower than 450 nM (24 h) and 200 nM (48 h) of celastrol. The tumor volume in the celastrol group was twice that in the NACC group. Importantly, a tissue distribution study indicated that NACC could selectively target tumor tissue and accumulation in liver and kidney tissues was decreased. The tissue distribution assay confirmed that nucleic acid aptamer modification could enhance anticancer activity and reduce liver toxicity (Liu, 2017).

Dendrimers are highly ordered, branched polymeric molecules with nano-sized, well-defined, homogeneous, and monodisperse structure consisting of branch-like arms (Srinivasa-Gopalan and Yarema, 2007). Dendrimers are hyperbranched macromolecules with a carefully tailored architecture, the end-groups (i.e., the groups reaching the outer periphery) of which can be functionalized modifying their biological and physicochemical properties (Smith and Diederich, 1998). The combination of nucleic acid aptamer technology and dendrimer technology can largely improve the targeting ability, thus enhancing efficacy and reducing toxicity.

**FIGURE 2:**
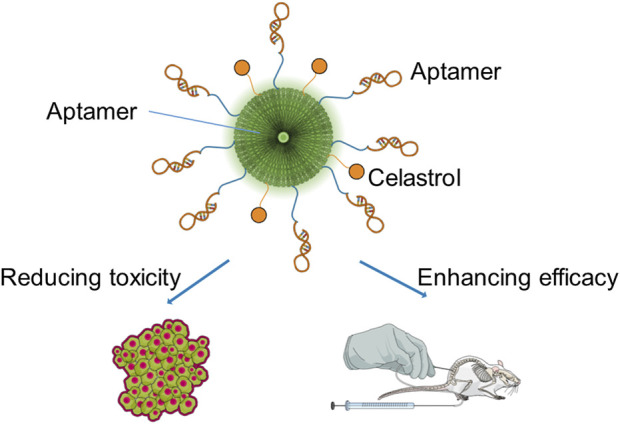
Schematic diagram of the smart conjugate with direct chemical connection of dendrimers and celastrol.

It has been reported that a smart conjugate composed of epithelial cell adhesion molecule (EpCAM) aptamer, dendrimer, polyethylene glycol (PEG), and celastrol specifically delivers celastrol into EpCAM-abundant tumor tissues to enhance the anticancer efficacy and reduce the toxicity. Figure 2 shows the schematic diagram of the smart conjugate with direct chemical connection of dendrimers and celastrol. Taking advantage of the increased water solubility and outstanding targeting ability of the aptamer and dendrimer, the smart aptamer–dendrimer–celastrol conjugate exhibited super apoptosis-inducing effects compared to free celastrol in EpCAM-positive colorectal cancer cell line SW620 both *in vitro* and *in vivo*. Importantly, the safety of the aptamer–dendrimer–celastrol conjugate experimented in animal models has indicated that the combination delivery system could remarkably reduce local and systemic toxicity (Ge et al., 2020).

Celastrol-loaded glucolipid-like conjugates with αvβ3-ligand tetraiodothyroacetic acid (TET) modification (TET-CSOSA-celastrol) were established to block the NF-κB signaling pathway. The system uses TET-CSOSA-celastrol to target and block the NF-κB signaling pathway. The distribution of TET-CSOSA was significantly increased in lung metastasis and primary tumor sites in 4T1 tumor-bearing mice due to αvβ3 receptor–mediated interaction. The study found that TET-CSOSA-celastrol effectively suppressed Bcl-2 activation in lung metastatic cells and reduced MMP-9 expression in 4T1 breast tumor cells. The inhibitory rates of TET-CSOSA-celastrol against lung metastasis and primary tumor increased to 90.72% and 81.15% when compared to that of celastrol (72.15% and 46.40%, respectively) (Zhao et al., 2018).

## 3 Indirect physical connection delivery system

**FIGURE 3:**
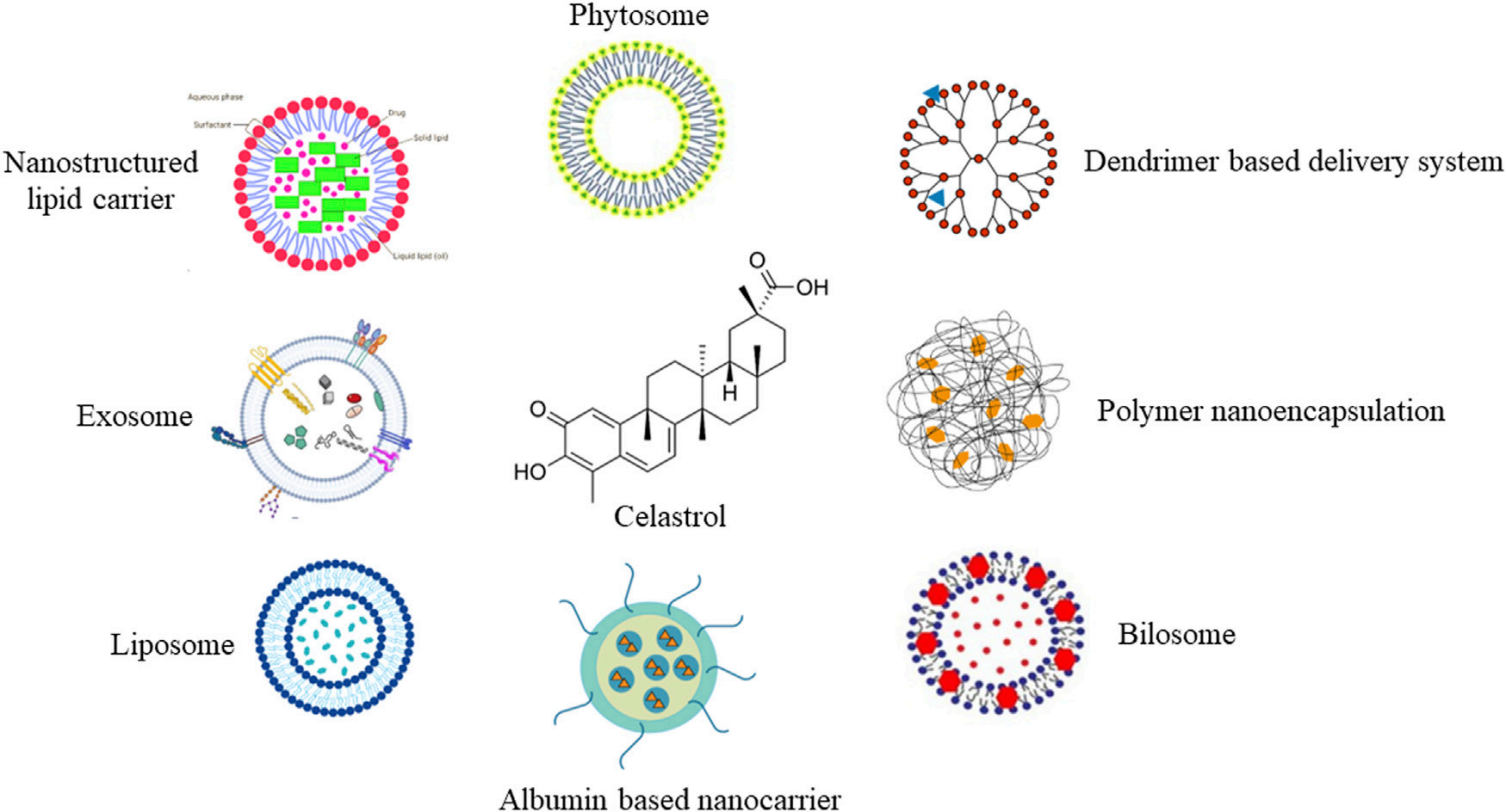
Summary figure of the indirect physical connection delivery system for celastrol.

Indirect physical connection delivery systems are drug delivery strategies that employ an indirect association between the therapeutic agent and a carrier system, without the requirement for covalent bonding or direct chemical modification. These systems often rely on non-covalent interactions, such as electrostatic forces, hydrophobic forces, or van der Waals forces, to facilitate the association between the drug and carrier. In this system, the therapeutic agent is encapsulated or embedded within the carrier structure, allowing for protection against degradation, improved solubility, controlled release, and enhanced bioavailability. This approach is particularly useful for celastrol to overcome various pharmacokinetic challenges. Indirect physical connection delivery systems can be tailored to achieve specific drug release profiles, targeting capabilities, and biodistribution patterns, making them versatile tools for a wide range of therapeutic applications. A summary is shown in Figure 3.

### 3.1 Dendrimer-based delivery system

**FIGURE 4:**
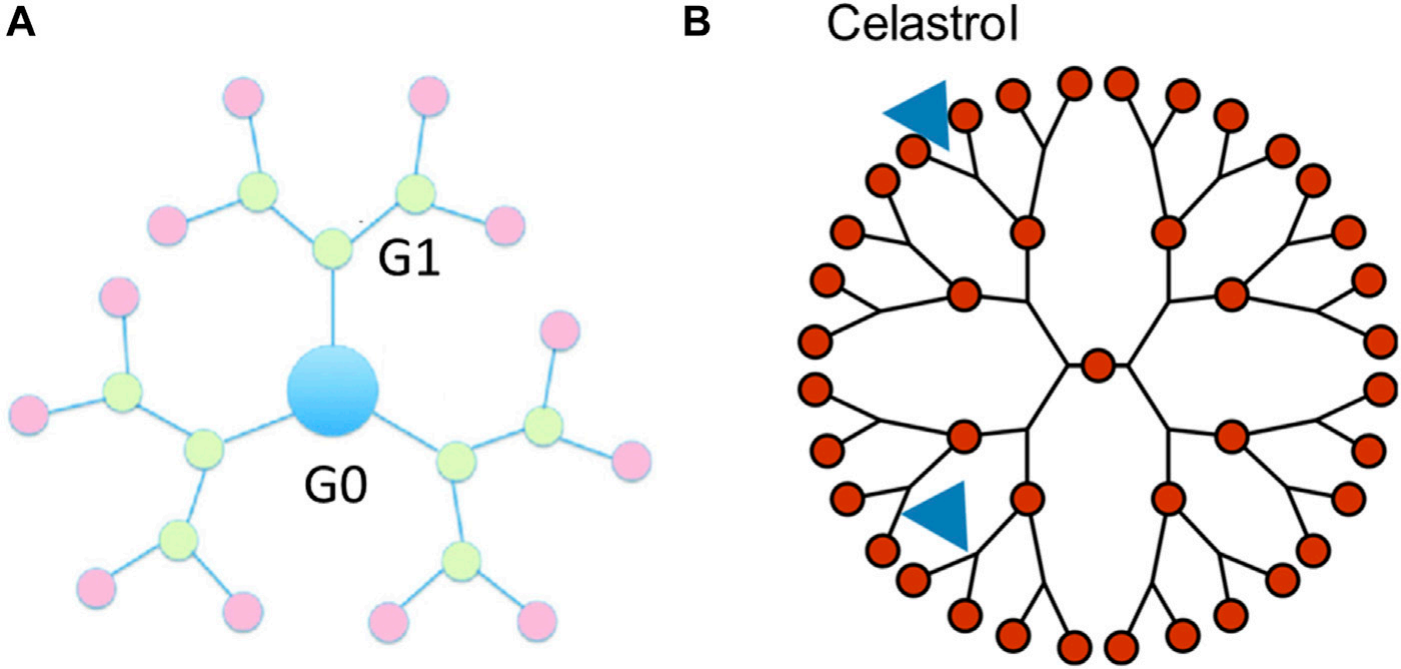
Schematic diagram of the celastrol-loaded dendrimers with the indirect physical connection delivery system. **(A)** Generations of dendrimers. **(B)** 2D Schematic of celastrol-loaded dendrimers.

A dendrimer is a highly defined artificial polymer macromolecule, which is characterized by a combination of a large number of functional groups and a compact molecular structure (Abbasi et al., 2014). The emerging role of dendrimer macromolecules for anticancer treatment is extraordinary. The advantages of these dendritic macromolecules make them a novel category of macromolecular nanoscale delivery systems (Wolinsky and Grinstaff, 2008). A number of active therapeutic compounds can be physically or chemically attached to the dendrimer (Chis et al., 2020). Up to now, poly(amidoamine) (PAMAM) dendrimers are the most reported with their surface groups that can be modified for the synthesis of anionic, cationic, or neutral dendrimers with increasing generations, as shown in Figure 4A.

It has been investigated that celastrol incorporated into PAMAM dendrimer macromolecules exhibited the capacity of a potent anti-inflammatory nanomedicine to inhibit endotoxin-mediated signaling in microglia, as shown in Figure 4B. Celastrol attaches to the PAMAM dendrimers by electrostatic interactions and hydrophobic interactions, which make an indirect physical connection. The water solubility of celastrol incorporated into the PAMAM dendrimer was remarkably increased. Celastrol conjugated to the amino termini of PAMAM dendrimers (celastrol/G4-NH2) showed stronger cytotoxicity in microglia than celastrol alone while exhibiting minimal anti-inflammatory activity. On the contrary, celastrol conjugated to the hydroxyl termini of PAMAM dendrimers (celastrol/G4-OH) could impede the release of pro-inflammatory cytokines without reducing microglial cell viability (Boridy et al., 2012). The above results support the possible usage of PAMAM dendrimers for effective anti-inflammatory therapy, which could enhance the anti-inflammatory efficacy.

### 3.2 Polymer nanoencapsulation-based delivery system

Nanoencapsulation is a technology encircling bioactive molecules in liquid or solid state in an inert material for preserving the coated molecules (Sanna et al., 2015). Nanoencapsulation of bioactive drugs in nanocarriers is a very prospective method for developing nanomedicine. The latest molecular encapsulation technique allows the effective loading of bioactive molecules inside the material, thus reducing local or systemic toxicity. The targeting ability of nanocarriers could enhance the accumulation of encapsulated bioactive molecules at the desired tissues (Kumari et al., 2014). Generally, most of the nanoencapsulation technology is based on the indirect physical connection, which encloses the bioactive molecules inside the packaging.

Recently, a celastrol-loaded poly(ε-caprolactone) nanocarrier system was developed using nanoprecipitation technique with remarkably higher anticancer activities against prostate cancer cell lines than celastrol control usage (Sanna et al., 2015). However, a simple celastrol-loaded poly (ε-caprolactone) nanocarrier showed nonspecific distribution in different tissues after injection and was easily trapped by the reticuloendothelial system, which intrinsically led to low anticancer properties *in vivo*. In order to obtain higher anticancer activity of celastrol, a reticuloendothelial system saturation method was performed. Empty nanocarriers were injected before the celastrol-loaded nanocarriers, which reduced the reticuloendothelial system’s ability to capture celastrol (Yin et al., 2017). The results showed that injection of empty nanocarriers without celastrol exhibited a longer blood circulation time for celastrol-loaded nanocarriers, and thus celastrol could specifically target to the tumor tissue. With an aim of achieving better selectivity, a neutrophil membrane–coated poly(ethylene glycol) methyl ether-block-poly(lactic-co-glycolic acid) (PEG-PLGA) nanocarrier system was designed by coating membranes of neutrophils on celastrol-loaded PEG-PLGA nanocarriers for target treatment of pancreatic cancer (Cao et al., 2019). Celastrol-loaded PEG-PLGA nanocarriers could target to the tumor tissue and specifically and remarkably decrease the tumor size of pancreatic ductal adenocarcinoma–bearing mice. Celastrol-loaded PEG-PLGA nanocarriers exhibited the longest survival time and minimum liver metastasis among the control groups. The above results indicate that celastrol-loaded PEG-PLGA nanocarriers represent a viable and effective treatment option for pancreatic cancer. Similar results and conclusions were reported in celastrol-loaded PEG-PLGA nanocarriers against malignant melanoma, with notably enhanced antitumor activity and apoptosis-inducing effect *in vitro* and *in vivo* (Zhou et al., 2019).

### 3.3 Albumin-based nanocarrier delivery system

Albumin is an attractive conveyor in delivering drugs of low water solubility. Firstly, albumin is the most plentiful protein in the plasma with high cellular biocompatibility, biodegradability, non-immunogenicity, and safety for its clinical application. Secondly, the molecular structure of albumin is such that it can interact with other diverse molecules, preventing elimination, thus enhancing molecular bioactivities. At last, albumin can interact with targets overexpressed in many cells and tissues, providing a delivery system for targeting to the desired position (Spada et al., 2021). Albumin-based nanocarriers can be produced by multiple approaches (Parodi et al., 2019) with various functions, such as specific targeting, drug delivery, and overcoming drug resistance (Maeda et al., 2000).

**FIGURE 5:**
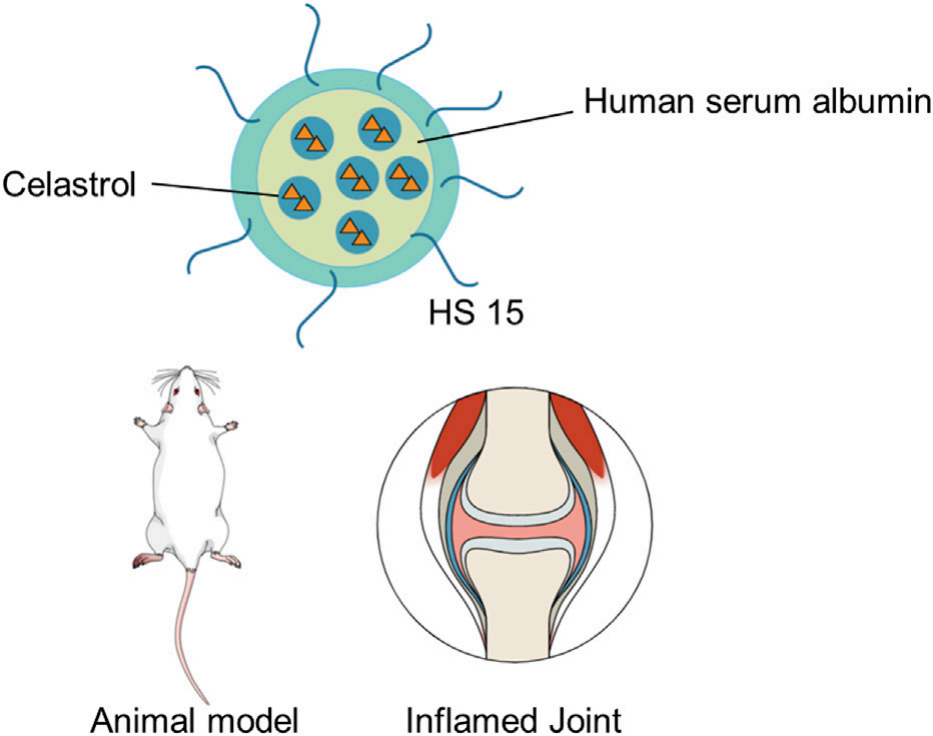
Schematic diagram of the celastrol-loaded human serum albumin nanocarriers.

Due to its hydrophobic feature and high affinity to albumin, celastrol is suitably enclosed into albumin-based nanocarriers. Recently, a microfluids co-flow method was proposed to produce celastrol-loaded human serum albumin nanocarriers with homogeneous nanocarrier size distribution by regulating the flow rate (Hakala et al., 2020). The celastrol-loaded albumin nanocarriers could improve water solubility of celastrol and reduce its cellular toxicity. Guo et al. (2017) encapsulated celastrol into albumin-based nanocarriers to deliver celastrol specifically to mesangial cells, thus accumulating to the mesangial tissue. The result showed that celastrol-loaded albumin nanocarriers performed better than celastrol alone in animal models. Moreover, celastrol-loaded albumin nanocarriers exhibited less celastrol accumulation in other tissues than celastrol alone, thus reducing local or systemic toxicity. A human serum albumin–Kolliphor^®^ HS 15 nanocarrier (HSA-HS15) system was constructed to address the limitations in targeted therapy for rheumatoid arthritis (RA) and enhance the safety of celastrol-loaded human serum albumin nanocarriers, as shown in Figure 5 (Gong et al., 2019). The result showed that celastrol-loaded HSA-HS15 exhibited notably higher treatment activity and safety in the treatment of RA than did free celastrol and celastrol-loaded human serum albumin nanocarriers. HSA-HS15 could be a safe and efficient therapeutic method for the treatment of RA.

### 3.4 Vesicular nanocarrier delivery system

Vesicular nanocarriers have received much attention in drug discovery and drug delivery, such as liposomes, exosomes, and nanostructured lipid nanocarriers. The vesicular nanocarrier delivery system can transfer hydrophilic and hydrophobic drugs with little toxicity, prolong the half-life time of drug action, and control the release of drugs (Ozpolat et al., 2014). The vesicular nanocarrier delivery system could be chemically modified to prevent identification by the immune system (PEG) and enhance the water solubility of drugs. Moreover, the vesicular nanocarrier delivery system could also be synthesized in a pH sensitive manner to improve drug release in an acidic microenvironment and be associated with antibodies or aptamers that identify the receptors (Rama et al., 2016).

The liposome technique was the first therapeutic carrier to be approved by the US Food and Drug Administration (FDA) to transport anticancer agents. Song et al. (2011) encapsulated the celastrol into liposomes to overcome the problem of low bioavailability. The result indicated that celastrol-loaded liposome exhibited higher absorption ability in intestinal tissue. In addition, oral celastrol-loaded liposome showed a higher tumor suppression rate in a lung cancer animal model than free celastrol. Chen et al. (2020a) constructed a novel celastrol-loaded liposome coated with galactose molecules binding with the asialoglycoprotein target to improve celastrol transport to the human liver cancer cell for therapeutic application . Celastrol-loaded galactosylated liposome increased the cellular uptake of celastrol by ligand–receptor interaction, thus increasing its anticancer activity against tumor cells *in vitro*. Celastrol-loaded galactosylated liposome treatment mainly reduced the damage to liver tissue and efficiently suppressed the growth of hepatocellular carcinoma by inhibiting AKT activation, inducing cell apoptosis and cell proliferation. In addition, celastrol-loaded galactosylated liposome caused some weight loss and harm to other tissues *in vivo*.

Exosomes can be widely found in all bodily fluids and tissues, such as the blood and urine (Caby et al., 2005). Exosomes are cell-derived nanovesicles that are involved in the intercellular transportation of materials. Therapeutic agents, such as small molecules or nucleic acid drugs, can be enclosed into exosomes and then transported to specific types of cells or tissues to accomplish drug delivery (Batrakova and Kim, 2015). Exosomes were extracted from bovine raw milk and then used for fast mixture manipulation conducted in the presence of ethanol to enclose celastrol into the exosomes (Aqil et al., 2016). Furthermore, the anticancer potential ability of the celastrol-loaded exosomes was tested in the lung cancer model. Celastrol-loaded exosomes showed a significantly higher anticancer activity against lung cancer cells than free celastrol. Celastrol provided significant inhibition (52% reduction) of tumor growth compared to vehicle treatment, and exo-celastrol showed significantly enhanced antitumor activity with 77% reduction presumably due to higher bioavailability of celastrol in the exosomal formulation. Additionally, oral celastrol-loaded exosomes inhibited the growth of lung tumor in xenografts of animal models efficiently. The above results have demonstrated that the administration of celastrol-loaded exosomes orally is a feasible method to efficiently inhibit the growth of lung tumors.

A nanostructured lipid carrier is a novel pharmaceutical delivery vector which is composed of biocompatible and physiological lipids, surfactants, and co-surfactants (Chauhan et al., 2020). Chen et al. (2012) constructed a topical nanostructured lipid carrier delivery system to improve the skin penetration for celastrol. The celastrol-loaded nanostructured lipid carrier led to the prolonged release property of celastrol and increased celastrol accumulation into the upper skin layer. In order to enhance the therapeutic effect of the celastrol-loaded nanostructured lipid carrier, the effect of the surface charge of the nanostructured lipid carrier on *in vitro* skin penetration and *in vivo* therapeutic efficacy of celastrol were carefully tested. The skin permeation result demonstrated that the cationic nanostructured lipid carrier delivered the most celastrol. The cationic nanostructured lipid carrier exhibited the highest cellular uptake and best cytotoxicity among the experimental groups in cell assays. Meanwhile, percutaneous administration of the cationic nanostructured lipid carrier showed the highest *in vivo* anticancer ability in the melanoma tumor model. The above results have indicated that the cationic nanostructured lipid carrier was a prospective delivery system of celastrol for topical anti-melanoma application.

Bilosomes are a novel type of lipid-based nanocarrier system that has been developed to improve the bioavailability and stability of various drug molecules (Waglewska et al., 2020). These vesicles are composed of bile salts, phospholipids, and cholesterol, which together form a bilayer structure that encapsulates the drug of interest. Using bilosome as a carrier for celastrol can potentially enhance the solubility, bioavailability, and targeted delivery of celastrol to specific tissues or cells (Yang et al., 2019). This approach may help overcome some of the challenges associated with celastrol's toxicity and poor water solubility, which limit its clinical application. By incorporating celastrol into bilosomes, researchers aim to develop a more effective and safer drug delivery system for treating various diseases, such as cancer and inflammatory disorders. Yang et al. (2019) developed hyaluronic acid (HA)–functionalized bilosomes for targeted delivery of *T. wilfordii* (Tri) to inflamed joints, aiming to enhance antiarthritic efficacy. HA@Tri-BLs were created by coating bilosomes with HA, resulting in small particle sizes, high entrapment rates, and sustained release of Tri. *In vivo* pharmacokinetic studies demonstrated improved circulation time and enhanced systemic and intra-arthritic bioavailability of Tri through HA@Tri-BLs. HA@Tri-BLs exhibited superior *in vivo* anti-inflammatory effects than HA-free Tri-BLs, likely due to HA-mediated transport that facilitated higher accumulation of Tri in the articular cavity. It highlighted the potential of using the HA ligand and flexible vesicles for targeted arthritis therapy and specific delivery of anti-inflammatory agents.

Phytosomes are a novel drug delivery system designed to enhance the absorption, bioavailability, and therapeutic efficacy of plant-derived compounds, especially those with poor solubility and absorption characteristics (Barani et al., 2021). They are formed by complexing phytochemicals with phospholipids, creating a lipid-compatible molecular complex that can easily cross biological membranes (Alharbi et al., 2021). The phytosome technology improves the pharmacokinetic profile of the encapsulated phytochemicals, allowing them to be absorbed more efficiently and distributed to target tissues more effectively. This results in an enhanced therapeutic effect and reduced side effects. Phytosomes have been employed to increase the bioavailability of various plant-derived compounds (Babazadeh et al., 2019). A study focused on the development of self-assembled phytosomal nanocarriers (celastrol-PHY) to improve the solubility and oral bioavailability of celastrol (Freag et al., 2018). The researchers prepared a celastrol–phospholipid complex using a solvent evaporation technique and confirmed its formation through various analytical methods. The optimized celastrol-PHY exhibited a nanometric particle size and negative zeta potential, which enhanced celastrol release compared to the crude drug and physical mixture. Pharmacokinetic studies in rabbits demonstrated a significant improvement in celastrol-PHY oral bioavailability compared to crude celastrol, with a fourfold increase in AUC0-8 and a fivefold increase in C_max_. The results highlighted the potential of phytosomal nanocarriers to improve celastrol oral delivery, opening new possibilities for its use in oral cancer therapy.

## 4 Discussions and conclusion

There are many ways to modify and deliver celastrol, as has been presented in this review, which include direct and indirect modification systems as well as specific modification and delivery methods. There are several advantages to direct modifications. Covalently attaching groups directly to celastrol allows precise control over physicochemical properties like solubility and stability. This can more reliably improve bioavailability. Direct targeting moieties like aptamers provide a more direct way to enhance tissue/cell specificity. However, there are also potential shortcomings of direct modifications. Covalent modifications can alter celastrol’s structure and biological activity. This has to be carefully evaluated. Synthetic procedures may be more complex than indirect encapsulation methods. There are several advantages to indirect modifications, where no chemical changes are made to celastrol, thereby reducing risks of altering its pharmacological properties. Various carriers provide versatility in properties like sizes, surface properties, and targeting abilities. Encapsulation protects celastrol and provides a controlled release. However, there are also potential shortcomings of indirect modifications. The release of celastrol depends on carrier degradation/dissolution and may not be easily controlled. Carrier properties and interactions with celastrol have to be thoroughly optimized. Targeting may depend more on carrier properties than on direct targeting moieties.

The modification methods of celastrol to enhance its therapeutic efficacy and reduce toxicity have primarily focused on direct modifications, such as structural alterations, and indirect strategies, which include the use of targeted delivery systems like nanoparticles. These strategies aim to improve celastrol’s bioavailability, increase its specificity toward pathological targets, and minimize off-target effects. For instance, encapsulating celastrol in nanoparticles has been shown to enhance its delivery to tumor sites, thereby improving its anticancer efficacy. Similarly, structural modifications of celastrol that reduce its toxicity can make it a safer option for treating chronic inflammatory diseases.

Although these modification methods have shown promise in diseases such as cancer and certain inflammatory conditions, their applicability and effectiveness in a broader spectrum of diseases remain under-explored. The types of diseases treated by these modification methods are relatively limited, partly due to the initial focus on conditions where celastrol’s mechanisms of action are most relevant, such as those involving inflammation and abnormal cell proliferation.

The question arises whether celastrol, with specific modifications, could also play a role in treating other diseases beyond the current focus areas. Given celastrol’s multifaceted biological activities, there is the potential for its application in diseases not traditionally associated with its known mechanisms of action. For example, neurodegenerative diseases, where inflammation and oxidative stress play critical roles, could benefit from treatment with celastrol’s neuroprotective and anti-inflammatory properties. Similarly, metabolic disorders that involve inflammatory processes might also be amenable to treatment with modified forms of celastrol.

Expanding the therapeutic use of celastrol to a wider range of diseases entails several challenges and considerations. Firstly, a thorough understanding of the disease pathology and how celastrol’s mechanisms of action intersect with these pathological processes is essential. This requires extensive preclinical research to identify potential therapeutic targets and understand the potential side effects and toxicity in the context of different diseases. Secondly, the development of modification methods that can tailor celastrol’s action to specific diseases is crucial. This involves not only enhancing its therapeutic efficacy but also ensuring that modifications do not compromise its beneficial properties or introduce new safety concerns. Lastly, clinical validation of modified celastrol’s efficacy and safety in a broader range of diseases is necessary. This step is critical for translating preclinical findings into clinical applications and requires well-designed clinical trials that consider the specificities of each disease.

In general, natural products have been served as a crucial and inestimable source of new drug development. Among them, celastrol is one of the most attractive molecules with a variety of potential pharmacological activities in diverse diseases, such as inflammatory, obesity, cancer, and bacterial diseases. The poor physicochemical features of celastrol are the main limitations during the progression of its development, and thus there are few advancements in clinical application for celastrol. In the past decades, a number of studies have been reported to address these issues by direct chemical modification and the indirect physical connection delivery system. We made a comprehensive review to light the way of celastrol modification, which could enhance the efficacy and reduce the toxicity of celastrol.
